# Paternally Expressed Imprinted Genes under Positive Darwinian Selection in *Arabidopsis thaliana*

**DOI:** 10.1093/molbev/msz063

**Published:** 2019-03-26

**Authors:** Reetu Tuteja, Peter C McKeown, Pat Ryan, Claire C Morgan, Mark T A Donoghue, Tim Downing, Mary J O’Connell, Charles Spillane

**Affiliations:** 1 Genetics & Biotechnology Lab, Plant & AgriBiosciences Research Centre (PABC), School of Natural Sciences, Ryan Institute, National University of Ireland Galway, Galway, Ireland; 2 School of Biotechnology, Faculty of Biological Sciences, Dublin City University, Dublin, Ireland; 3 Computational and Molecular Evolutionary Biology Research Group, School of Biology, Faculty of Biological Sciences, The University of Leeds, Leeds, United Kingdom; 4 Computational and Molecular Evolutionary Biology Group, School of Life Sciences, University of Nottingham, Nottingham, United Kingdom; 5 Center for Genomics and Systems Biology, New York University, New York, NY; 6 Division of Diabetes, Endocrinology and Metabolism, Imperial College London, London, United Kingdom; 7 Memorial Sloan Kettering Cancer Center, New York, NY

**Keywords:** genomic imprinting, genomic conflict, positive Darwinian selection, endosperm, plant evolution

## Abstract

Genomic imprinting is an epigenetic phenomenon where autosomal genes display uniparental expression depending on whether they are maternally or paternally inherited. Genomic imprinting can arise from parental conflicts over resource allocation to the offspring, which could drive imprinted loci to evolve by positive selection. We investigate whether positive selection is associated with genomic imprinting in the inbreeding species *Arabidopsis thaliana*. Our analysis of 140 genes regulated by genomic imprinting in the *A. thaliana* seed endosperm demonstrates they are evolving more rapidly than expected. To investigate whether positive selection drives this evolutionary acceleration, we identified orthologs of each imprinted gene across 34 plant species and elucidated their evolutionary trajectories. Increased positive selection was sought by comparing its incidence among imprinted genes with nonimprinted controls. Strikingly, we find a statistically significant enrichment of imprinted paternally expressed genes (iPEGs) evolving under positive selection, 50.6% of the total, but no such enrichment for positive selection among imprinted maternally expressed genes (iMEGs). This suggests that maternally- and paternally expressed imprinted genes are subject to different selective pressures. Almost all positively selected amino acids were fixed across 80 sequenced *A. thaliana* accessions, suggestive of selective sweeps in the *A. thaliana* lineage. The imprinted genes under positive selection are involved in processes important for seed development including auxin biosynthesis and epigenetic regulation. Our findings support a genomic imprinting model for plants where positive selection can affect paternally expressed genes due to continued conflict with maternal sporophyte tissues, even when parental conflict is reduced in predominantly inbreeding species.

## Introduction

Rapid evolution under Positive Selection (PS) is a feature of many reproductive proteins in both plants and animals, occurring either as a result of adaptive radiation or of sexual conflict within and between genomes (Clark et al. 2006). For example, tests of selective pressure have shown that genes expressed in the highly reduced male gametophyte of flowering plants (the pollen grain) display elevated PS (Arunkumar et al. 2013; Gossmann et al. 2014). These increased levels of PS are observed in genes expressed in the pollen tube but not the sperm cell, and are interpreted to be a consequence of conflict driven by competition between pollen grains for access to ovules (Bernasconi et al. 2004). Conflict is also expected to occur at loci regulated by genomic imprinting, in which genes are monoallelically expressed under epigenetic regulation in a parent-of-origin specific manner, in violation of the Mendelian rules of genetic inheritance (Haig 1997; Wilkins 2011). Indeed, genomic imprinting is widely considered to have evolved due to conflict between parentally derived genomes over resource allocation to developing offspring which lead to genes evolving different optimal expression levels depending upon whether they are maternally- or paternally derived (Willson and Burley 1983; Wilkins and Haig 2003b; Haig 2004). Imprinting has been reported from both mammals and flowering plants, in which it principally occurs in the endosperm (Gehring and Satyaki 2017), the second product of double fertilization which provides maternally derived resources to the developing embryo in the seed (Walbot and Evans 2003). Imprinting leads to the occurrence of imprinted maternally expressed genes (iMEGS) and imprinted paternally expressed genes (iPEGS) (Haig and Westoby 1991; Garnier et al. 2008; Köhler et al. 2012). Kin conflict between iPEGs and iMEGs in plants is expected to arise from differences in the optimal level of offspring resource allocation, and resulting offspring size, between the maternal and paternal genomes as selection on the maternal genome favors equal provision to all offspring (and iMEGs near-equal provision; see Trivers 1974) while the paternal genome promotes growth of its own offspring alone (Haig 2000, 2013; Costa et al. 2012; Willi 2013).

**Fig. 1.**
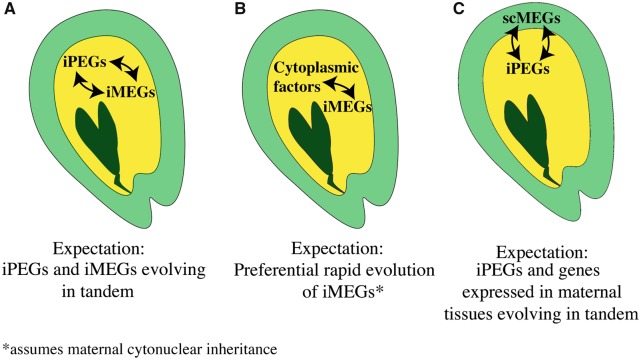
Summary of scenarios for selection on imprinted plant genes. Schematic of *Arabidopsis thaliana* seed summarizing the impacts of genomic imprinting on genetic selection as predicted by major hypotheses for genomic imprinting. In each case, the diploid F1 embryo is shown in dark green, surrounded by the triploid F1 endosperm, shown in yellow) in which imprinting occurs, and the diploid seed coat (SC) which is part of the maternal sporophyte, shown in light green. (*A*) Intragenomic conflict in which antagonism between matrigenes and patrigenes over resource allocation results in physical interactions between iMEGs and iPEGs (Spillane et al. 2007). (*B*) Coadaptation models predict that any selective pressure should be concentrated on iMEGs which are coinherited with cytoplasmic genomes in *A. thaliana* (Wolf and Brandvain 2014). (*C*) Indirect conflict or “Kinship Model” predicts that conflict between iPEGs and genes expressed in maternal tissues (e.g., seed coat, scMEG, or other sporophyte tissues) leads to positive selection on iPEGs (Willi 2013).

Such conflict can have different consequences at the molecular level, including conflict relating to expression level and rapid evolution of nucleotide sequence (or epigenetic signatures) associated with gene expression (Haig et al. 2014). At the level of the coding sequence, one prediction is that conflict can lead to positive selection on pairs of reciprocally imprinted genes expressed from the maternally and paternally inherited genomes, each having antagonistic effects on offspring growth (Wilkins and Haig 2001; Mills and Moore 2004). We illustrate this occurring inside the endosperm of the seed (yellow) in figure 1*A*, within which iMEGs and iPEGs mutually interact. Some support for this particular form of parental conflict has been found in mammals, for example, at the *Igf-2* and *callipyge* loci (Georges et al. 2003; Reik et al. 2003; Crespi and Semeniuk 2004). Signatures of positive selection have also been detected at the imprinted *MEDEA* locus in the flowering plant *Arabidopsis lyrata* (Spillane et al. 2007; Miyake et al. 2009) which may support the hypothesis that imprinting can cause positive selection on coding sequences of the loci concerned. On the other hand, conflict can have other molecular effects, including selection for stable equilibria of iMEG and iPEG expression levels (Haig 2014), and coevolutionary scenarios between iMEGs and cytoplasmic factors (Wolf and Hager 2006), as shown in figure 1*B*. It has also been suggested that conflict could occur between iPEGs and the tissues of the maternal sporophyte (Willi 2013): the genes of the seed coat (SC) are also maternally derived and could therefore act in a manner antagonistic to iPEGs—this scenario of “indirect conflict” between the genes of the maternal seed coat (which we denote scMEGs) and iPEGs in the endosperm is shown in figure 1*C*. It has been alternatively suggested that imprinting in plants could be related to the biology of gene expression in triploid endosperm, for example, as a dosage control mechanism, although a recent study of gene expression in triploid embryos did not support this (Fort et al. 2017).

Genomic imprinting also occurs in the model plant, *Arabidopsis thaliana* (L.) Heynh, which is the sister species to *A. lyrata*, at *MEDEA* and several hundred other loci (Gehring et al. 2011; Hsieh et al. 2011; McKeown et al. 2011; Wolff et al. 2011). Furthermore, a subset of imprinted genes which are expressed early in *A. thaliana* seed development (4 days after pollination) display accelerated evolutionary rates compared with nonimprinted genes (Wolff et al. 2011) as measured by *D*_N_/*D*_S_. The rate of nonsynonymous mutations per nonsynonymous site (*D*_N_) and the rate of synonymous mutations per synonymous site (*D*_S_) is assumed to follow the neutral evolutionary process and the ratio, such that *D*_N_/*D*_S_ (also denoted ω), is therefore approximate to the selective pressure on the protein product of a gene. A value of *ω *> 1 signifies positive selection (PS) at a site, *ω *≈ 1 implies neutral evolution, while *ω *< 1 indicates purifying selection. It should be noted that positive selection typically only acts at a subset of amino acid sites while other sites are typically still under purifying selection, so *ω* is still generally <1 at the level of the whole gene even when PS has occurred. Hence, comparisons between sets of candidate genes and relevant control sets are needed to identify elevated levels of *ω.* Enrichment for sites with *ω *> 1 in the data set of Wolff et al. (2011) when compared with controls in this way was therefore interpreted as a possible signature for conflict-driven selection within plant imprinted genes.

Evidence of elevated rates of adaptive substitution has also been reported for imprinted genes of the outcrossing Brassicaceae species, *Capsella rubella* (Hatorangan et al. 2016). This suggests that increased PS could be a general phenomenon for imprinted genes, supporting models of the parental conflict theory in which conflict leads to rapid evolution of coding sequences. However, it is important to note that elevated *D*_N_/*D*_S_ values can be caused by other factors such as variable effective population size, *N*_e_ (Kryazhimskiy and Plotkin 2008; Jensen and Bachtrog 2011) and selection on silent sites (Chamary et al. 2006). It is also unclear whether potential PS in *A. thaliana* or *C. rubella* is acting equally on iMEGs or iPEGs as would be consistent with models of parental conflict involving direct interactions between the proteins which they encode (fig. 1*A*): iMEGs and iPEGs both showed higher *D*_N_/*D*_S_ in the study of (Wolff et al. 2011), although in *C. rubella* increased accumulation of nearly neutral nonsynonymous variants was restricted to iPEGs (Hatorangan et al. 2016). Nor has it been shown whether past positive selection has led to fixation within current plant populations, as would be expected if the selection acting on amino acids is functionally significant for protein function.

To determine whether genomic imprinting in the seed endosperm is associated with positive selection in plant genomes, we analyzed the selective pressures acting on a comprehensive group of all confirmed imprinted genes of *A. thaliana* (Gehring et al. 2011; Hsieh et al. 2011; McKeown et al. 2011; Wolff et al. 2011). Specifically, we addressed the following questions: 1) What selective pressures are imprinted genes evolving under in *A. thaliana*? 2) If imprinted genes are evolving under positive selection, does this lead to overall positive selection in iMEGs and/or iPEGs being elevated compared with similar sets of biallelically expressed genes? And 3) Is there evidence for fixation of positively selected sites in imprinted genes across sequenced *A. thaliana* accessions? Our findings in relation to these questions extend our understanding of the evolutionary drivers of genomic imprinting and the consequences of parental conflict during reproduction.

## Results

### Imprinted *Arabidopsis thaliana* Genes Are Rapidly Evolving

Genomic imprinting has been predicted to evolve due to parental conflicts over provision of maternal resources to offspring, which has been hypothesized to lead to positive selection at loci involved in this conflict. The model eudicot *Arabidopsis thaliana* has been reported to display genomic imprinting on at least 436 genes in its seed endosperm (Gehring et al. 2011; Hsieh et al. 2011; McKeown et al. 2011; Wolff et al. 2011), with growing consensus over a core set which appear to be stably imprinted in many accessions (Gehring and Satyaki 2017; Schon and Nodine 2017; Wyder et al. 2019). The identification of genes subject to monoallelic expression in the seed endosperm can be confounded by parent-of-origin specific expression patterns that can also arise during early seed development from gametophytic deposition of mRNA in the fertilized egg cell (zygote) or fertilized central cell (endosperm), or from maternal-expression from genes expressed in the sporophytic seed coat, which may be present as contaminants during RNA-seq analyses. To determine the selective pressures acting on imprinted genes, while avoiding these confounding scenarios, we focused our analyses on those genes with strong evidence for uniparental expression in seeds due to imprinting. We classified these as genes identified from RNA-seq-based studies (Gehring et al. 2011; Hsieh et al. 2011; Wolff et al. 2011) which are expressed from the paternal genome (iPEGs), and which therefore cannot be due to contamination from maternal tissues; and those iMEGs for which experimental validation of monoallelic expression and/or epigenetic regulation in the endosperm has been performed in planta (Vielle-Calzada et al. 1999; Kinoshita et al. 2004; Köhler et al. 2005; Tiwari et al. 2008; Gehring et al. 2009; Hsieh et al. 2011; McKeown et al. 2011; Shirzadi et al. 2011; Wolff et al. 2011). This produced a set of 140 high-confidence imprinted genes (supplementary table S1*A* and *B*, Supplementary Material online) of which 63 were iPEGs and 77 were iMEGs. By comparing the *A. thaliana* and *A. lyrata* orthologs, we determined that both iPEGs and iMEGs within the 140 imprinted genes had mean values of ω significantly higher than that of the background representing all other remaining *A. thaliana* genes (table 1; *U* test: iPEGs *P *=* *9.9e-07, iMEGs *P *=* *1.9e-06). This provides large-scale empirical evidence that rapid evolution previously observed in imprinted genes detected in seed offspring at 4 days after pollination from one set of reciprocal crosses (Wolff et al. 2011) applies more generally to the imprinted genes of *A. thaliana*.

**Table 1.** *D*_N_/*D*_S_ Ratios (*ω*) of iPEGs and iMEGs Compared with Whole Genome.

Gene Class	Mean *ω* (*D*_N_/*D*_S_)	Median *ω* (*D*_N_/*D*_S_)
iPEGs	0.4265±0.053	0.3339
iMEGs	0.5045±0.061	0.3314
Whole genome	0.2436±0.002	0.1814

### Imprinted Genes Are Evolving under Positive Selection in *A. thaliana*

**Fig. 2.**
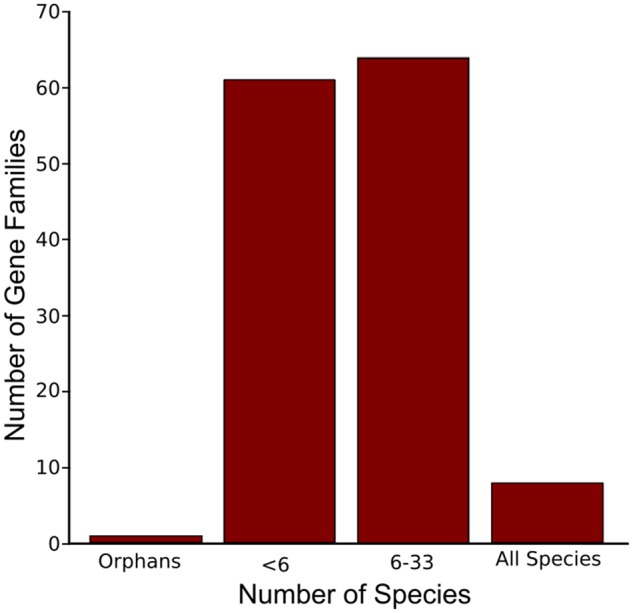
Size of orthology clusters to which imprinted *Arabidopsis thaliana* genes belong. Orphans are defined according to (Donoghue et al. 2011); genes present in orthology clusters >6 were considered for further selective pressure variation analysis.

PS can be detected at the population genomic level by assessing allele frequency and coalescence time as variation subject to PS is expected to go to fixation (Nielsen 2005; Sabeti et al. 2006). Genes can display elevated *ω* for a range of reasons other than PS, however, such as reduced functional constraint or pseudogenization. To test whether the increase in *ω* observed across the imprinted iMEGs and iPEGs was due to positive selection, we analyzed the evolutionary rates of iMEGs and iPEGs in the context of clusters of orthologous genes from across the plant kingdom. This analysis was conducted using an in-house plant database containing ortholog clusters from 34 sequenced plant species, either Embryophyte or Chlorophyte (supplementary fig. S1, Supplementary Material online). To further ensure the robustness of our analysis, we only considered clusters for which orthologous genes could be identified from at least six species, in addition to *A. thaliana* (see Materials and Methods), following recommended best practice for PAML analyses derived from simulation studies (Anisimova et al. 2001). Applying this filter, suitable clusters for PAML (codeML) analyses were obtained for 64 of the 140 imprinted genes (30 iMEGs and 34 iPEGs; fig. 2 and supplementary table S1*B*, Supplementary Material online). Sequence alignment quality is also critical for correct sequence analysis (Markova-Raina and Petrov 2011) so all alignments were also assessed using the norMD score as a proxy for alignment quality (Thompson et al. 2001)—see Materials and Methods for details. Two genes (iPEG AT4G11400, iMEG AT5G53870) that had poor sequence alignment quality (norMD score <0.6) were excluded from further analyses.

**Fig. 3.**
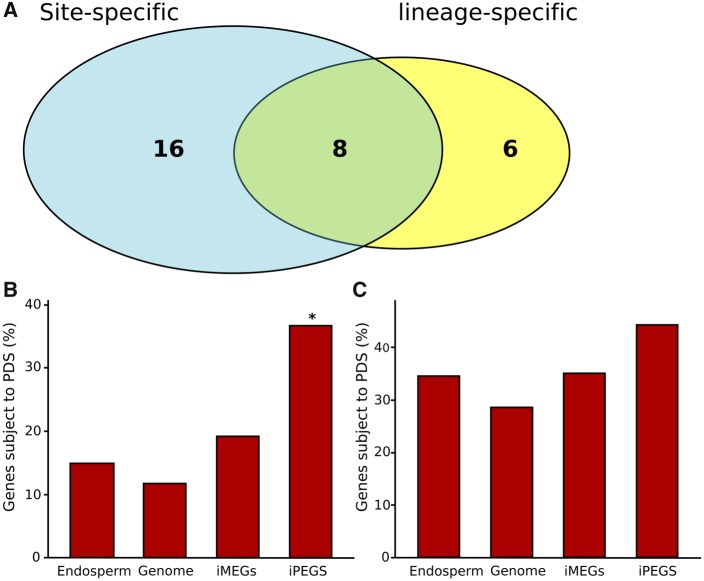
Summary of the number of genes under positive selection in the data set. (*A*) Numbers of imprinted *Arabidopsis thaliana* genes under site and/or lineage specific PS; (*B* and *C*) the percentages of *A. thaliana* iMEGs and iPEGs subject to lineage-specific (*B*) or site-specific (*C*) PS compared with the percentages in control sets of endosperm-expressed (“Endosperm”) or genome-wide (“Genome”) biallelic genes; control gene-sets are listed in supplementary table S4, Supplementary Material online.

Applying standard codeML models to the remaining 62 imprinted genes, we identified 30 that are evolving under PS (table 2 and fig. 3*A*;supplementary table S1, Supplementary Material online). For 6 of the 30 positively selected imprinted genes, the PS was specific to the *A. thaliana* lineage (i.e., lineage-specific PS; supplementary table S1*A*, Supplementary Material online), while for 16 imprinted genes positive selection was detected at individual codons in cross-lineage comparisons (i.e., site-specific PS, supplementary table S1*A*, Supplementary Material online). Eight imprinted genes displayed both lineage-specific and site-specific PS (fig. 3*A*). To ensure that these results have not been biased by any of the assumptions inherent in PAML, we also performed a HyPhy analysis (Pond and Muse 2005) on these 62 genes, using a combination of FEL (Fixed Effects Likelihood), SLAC (Single-Nucleotide Ancestor Counting), and MEME (Mixed Effects Model of Evolution) packages, as described in the Materials and Methods. From these analyses, we determined that PS is also predicted to be occurring on all 30 genes identified by PAML (supplementary table S2, Supplementary Material online). HyPhy and codeml-based models such as PAML differ fundamentally in how they estimate site-specific rates: PAML models use random effects likelihood while HyPhy models use fixed-effects likelihood, hence the congruence between the results of the two approaches provides strong confirmation of the robustness of the PS signature at the 30 imprinted loci.

**Table 2.** Numbers of iMEGs and iPEGs Determined to be under Positive Selection.

	iMEGs	iPEGs	Total
Total number of genes tested	30	**32**	**62**
Genes subject to lineage-specific selection only	2 (6.7%)	4 (12.5%)	**6 (0.9%)**
Genes subject to site-specific selection only	7 (23.3%)	9 (28.1%)	**16 (25.8%)**
Genes subject to both lineage- and site-specific selection	2 (6.7%)	6 (18.8%)	**8 (12.9%)**
Total	**11** (36.7%)	**19** (59.4%)	**30 (48.4%)**

Recently, a methodology has been published for directly estimating possible confounding of imprinting gene analysis by contamination with maternal tissues (Schon and Nodine 2017). Two of the data sets, of Gehring et al. (2011) and Hsieh et al. (2011), were analyzed by Schon and Nodine who suggested that 20 iMEGs from these studies used in our analysis should be considered “low-confidence” (although variation in gene expression patterns under different growth conditions could itself confound these conclusions). The RNA-seq data set of Wolff et al. (2011) was not analyzed by the Schon and Nodine (2017), so we performed the tissue-enrichment test of Schon and Nodine on the data sets used by Wolff et al. (2011) to determine expression pattern (Belmonte et al. 2013). We conclude that these data sets do not suffer from significant levels of cross-tissue contamination (supplementary fig. S2, Supplementary Material online): only the suspensor showing any potential contamination from nonsuspensor specific transcripts while none of the endosperm data sets used to identify imprinted genes showed any enrichment for other tissues, including the maternal seed coat. We conclude that the remaining 57/77 iMEGs used in our PAML and HyPhy analyses are “high-confidence” imprinted genes, while a further 20 may be due to the presence of maternally derived transcripts (supplementary table S3, Supplementary Material online). These include four genes which are under positive selection according to both codeML and HyPhy, ten others which showed no evidence for PS and six which were not tested due to lack of sufficient orthology clusters. We conclude that positive selection acts upon 19 iPEGs and 11 iMEGs, and that all of the iPEGs and at least 7 of the iMEGs are high-confidence imprinted genes. Taken together, these results indicate that positive selection acts on protein-coding genes regulated by genomic imprinting in the seeds of *A. thaliana*.

### Imprinted Genes Are Preferentially Affected by Positive Selection

The large number of imprinted genes subject to positive selection suggested that genes epigenetically regulated by genomic imprinting could be under stronger positive selection than biallelically expressed genes. To test this hypothesis, we compared the extent of positive selection in imprinted genes to that observed in randomly sampled gene sets from across the whole genome (supplementary table S4*A*, Supplementary Material online). Genomic imprinting in plants mainly occurs in the seed endosperm, which can be subject to different selective pressures related to its triploid genome dosage independent of imprinting (Baroux et al. 2002). Hence, we also conducted analysis of positive selection for random samples of known endosperm-specific *A. thaliana* genes (Belmonte et al. 2013) (supplementary table S4*B*, Supplementary Material online). For iPEGs, the odds ratio score for lineage-specific positive selection indicated 3.3- and 2.6-fold enrichment in positive selection in imprinted genes compared with whole-genome and endosperm controls, respectively. These ratios equate to a significant enrichment of lineage-specific positive selection in iPEGs when compared with either the genome-wide or endosperm-specific controls (Fisher’s test, *P *=* *0.014 and *P *=* *0.041 respectively; fig. 3*B*). Strikingly, no enrichment was found for iMEGs in either lineage-specific (*P *=* *0.531 vs. genome-wide controls, *P *=* *0.688 vs. endosperm genes) or site-specific selective pressure variation (*P *=* *0.542 vs. genome-wide controls, *P *=* *0.764 vs. endosperm genes) (fig. 3*C*), whether lower-confidence iMEGs were included or not. To determine if the bias in enrichment of position selection in iPEGs as compared with iMEGs is due to statistical threshold effect, we identified an additional set of imprinted genes where the significance level following LRT fell just below the cut off *P* value of 0.05 (but >0.10): out of the set of six imprinted genes identified with this relaxed criteria, only one imprinted gene is annotated as an iMEG, while the other five were iPEGs, therefore, we can discount any potential bias of this results due to thresholding. We further tested the strength of the difference between the selective pressures acting on iMEGs and iPEGs by performing a χ^2^ test directly on the ω-values as extracted from the branch site models (using likelihood ratio tests values from Morgan et al. 2010). We conclude that iPEGs, but not iMEGs, are subject to higher levels of positive selective pressure, revealing a difference in the evolutionary trajectory of imprinted genes depending on the parental genome from which they are expressed.

### Most Imprinted Genes Exhibit Fixation of Positively Selected Sites

If the sites determined to be under positive selection in the *A. thaliana* lineage improved plant fitness, then we could expect that these substitutions would be fixed or exist at high frequency within *A. thaliana* populations due to full or partial selective sweeps (Patwa and Wahl 2008). Hence, we tested the percentage conservation of *A. thaliana*-specific amino acid sites under either lineage-specific PS or site-specific PS (supplementary tables S5 and S6, Supplementary Material online). For almost all imprinted genes subject to lineage-specific PS, the associated sites showed 100% conservation across the 80 *A. thaliana* accessions for which full sequence data were available (posterior probability >0.95) (supplementary table S7, Supplementary Material online) (Cao et al. 2011), with no difference observed between iMEGs and iPEGs. Only two imprinted genes (AT1G48910 and AT1G55050) displayed nonsynonymous mutations at the otherwise conserved positively selected position. AT1G48910 encodes YUCCA 10, which is a flavin monooxygenase involved in auxin biosynthesis predicted to have roles in morphogenetic development of pollen grains, while AT1G55050 is a widely conserved gene of unknown function. If variation at the amino acids subject to positive selection confers phenotypic effects, this requires distinct *A. thaliana* populations with known population histories to test for differing intraspecific selection signatures driven by local environments (Huber et al. 2014). We consider that positive selective pressures at imprinted loci in the *A. thaliana* lineage has been sufficiently strong, (i.e., with a selective advantage for these alternative amino acids), to cause the fixation of these amino acid variants.

### Positive Selection on the Imprinted *NRPD1a* Gene Involved in sRNA Regulation

We noted that the imprinted genes subject to lineage-specific positive selection included *NRPD1a*, which encodes a component of the RNA Pol IV complex response for transcribing small RNA and, subsequently, transcriptional balance between maternally and paternally inherited genomes in endosperm (supplementary table S8, Supplementary Material online) (Kanno et al. 2005; Eamens et al. 2008; Erdmann et al. 2017). It has previously been reported that nucleotide substitution rate of the Pol IV polymerase subunit encoded by *NRPD1a* is 20 times higher than that observed in the equivalent subunit of Pol II (Luo and Hall 2007), supporting a scenario whereby the *NRPD1a* gene is under positive selection and suggesting a possible functional relationship between sRNA processing and (imprinted) genes under positive selection. We assessed if positive selection at *NRPD1a* might be due to selection occurring more generally on sRNA-processing genes, perhaps because of their roles in controlling the balance of maternal and paternal gene expression, and not due to the imprinting status of this gene specifically. However, when we analyzed the selective pressures acting on 23 nonimprinted genes encoding components of the sRNA processing pathway, none displayed any signature of positive selection (supplementary table S8, Supplementary Material online). We consider that the positive selection acting on *NRPD1a* is associated with its status as an imprinted gene involved in small RNA production and, likely, with subsequent control of gene expression in the endosperm.

### iMEGs and iPEGs Have Similar Evolutionary Ages

**Fig. 4.**
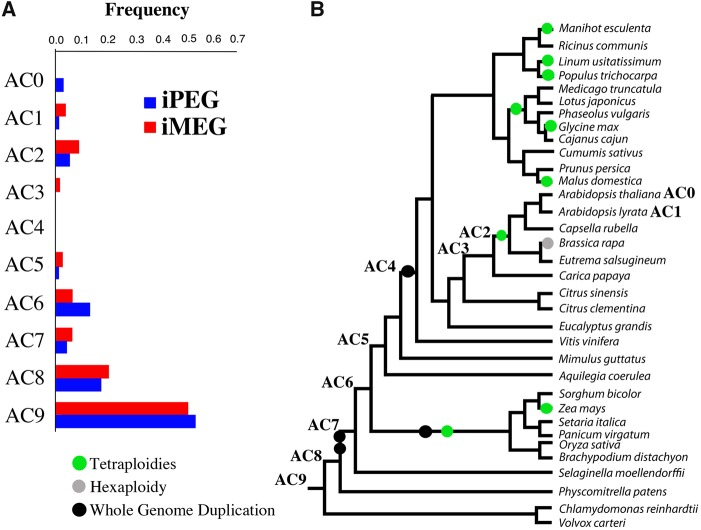
Phylogeny of the 34 species included in our analyses and the age distribution of iMEGs and iPEGs. (*A*) This shows the frequency of age class (AC) for the iMEGs and iPEGs tested. AC0, *Arabidopsis thaliana* specific; AC1, *A. lyrata*; AC2, Brassicaceae; AC3, Brassicales-Malvales; AC4, Rosid; AC5, Eudicot; AC6, Angiosperm; AC7, Tracheophyte; AC8, Embryophyte; AC9, Viridiplantae. (*B*) Consensus phylogenetic relationships of all 34 species; the phylogenetic position of the age classes and the known whole genome duplication events for the species included in the study are also highlighted (Vanneste et al. 2014).

One potential confounding factor in our analysis would be if iMEGs and iPEGs had different evolutionary ages. To address this possibility, we determined the evolutionary ages of the 140 imprinted genes using a phylostratigraphy approach (Domazet-Loso et al. 2007) (fig. 4). Nine Age Classes (AC) were defined for available plant genome sequences (https://phytozome.jgi.doe.gov/pz/portal.html; last accessed July 2015) where AC 0 includes the youngest genes (i.e., those which have evolved since the divergence of *A. thaliana*) and AC 9 the oldest, or most conserved. We then assigned imprinted genes to different age classes using an e-value cutoff of <10^−3^ (supplementary table S9, Supplementary Material online). Notably, no significant difference was observed between the age distributions of iMEGs and iPEGs (Fisher’s exact test, *P *=* *0.7), suggesting that differences in age are unlikely to explain the differing levels of PS observed in these categories.

Interestingly, 11 of the imprinted *A. thaliana* genes have been shown to have homologs regulated by imprinting in the sister species, *A. lyrata* (supplementary table S1*A*, Supplementary Material online), according to the analysis of (Klosinska et al. 2016). These include three iMEGs and eight iPEGs, including three iPEGs which we find to be under PS; these three all belonged to the most conserved age classes (8 or 9; supplementary table S9, Supplementary Material online) so may be good candidates for highly conserved imprinting. In contrast, a total of seven imprinted genes did not show any sequence similarity outside Brassicaceae (fig. 4), that is, they were Brassicaceae-specific orphans according to our previous definition (Donoghue et al. 2011). Of these Brassicaceae-specific imprinted orphan genes, one (AT4G31060) was found in *A. thaliana* only and so represents the most recently arisen imprinted gene known for this species. The fact that some imprinted genes date from the evolution of the angiosperms may indicate roles for these genes in the accompanying double fertilization event by which the endosperm evolved (Gehring et al. 2011), although this remains to be tested.

We found that the imprinted gene set as a whole showed enrichment for participation in the *At*-α whole genome duplication (WGD; 52 imprinted genes, Fisher’s test, *P *=* *0.02), whereas only 21 genes were found to have participated in either the *At-*β or *At-*γ WGD events (Fisher’s test, *P *=* *0.14) (fig. 4). The *At*-α WGD predated the diversification of core Brassicaceae from *Aethionema* (Franzke et al. 2011), while *At-*β and *At-*γ are older WGD events predating the emergence of Brassicaceae within the Eurosids (Bowers et al. 2003). These findings are in agreement with the models of Qiu et al., who suggested that many imprinted genes are descended from loci formed by WGD during the evolution of Brassicales (Qiu et al. 2014). However, there was again no difference in this distribution between iMEGs and iPEGs across different WGD events. In summary, we found no evidence for differing evolutionary histories or recent iPEG diversification that could confound our molecular evolutionary comparison between iPEGs and iMEGs.

### Most Imprinted Genes Are Functionally Constrained

Even if imprinted genes have been subject to positive selection in their evolutionary histories, it is possible that their recent evolution has been more constrained, for example, by purifying selection. To estimate the relative roles of ancestral PS (i.e., predating the most recent common ancestor of *A. thaliana* and *A. lyrata*) PS and recent selective constraint, we performed McDonald–Kreitman tests (McDonald and Kreitman 1991) on our entire set of 140 imprinted orthologs from *A. lyrata* and *A. thaliana* (this included the imprinted genes for which orthologs were identified in fewer than six other plant species, and which we had not been able to analysis by PAML or HyPhy). Unambiguous *A. lyrata* orthologs were detected for 110 out of the 140 total imprinted *A. thaliana* genes (56 iPEGs and 54 iMEGs) on the basis of BLASTP alignments (supplementary table S10*A*, Supplementary Material online). This approach assumed that the number of substitutions fixed between *A. thaliana* and *A. lyrata* was driven by ancestral positive selection and neutral substitution at nonsynonymous sites (*D*_N_), and by neutral processes only at synonymous ones (*D*_S_). As a result, a large *D*_N/_*D*_S_ ratio may indicate PS. We compared these *D*_N_ and *D*_S_ counts to the numbers of nonsynonymous (*P*_N_) and synonymous (*P*_S_) polymorphisms within the population of 80 genome-sequenced *A. thaliana* accessions to determine the fixation index (*FI*) such that *FI*=(*D*_N_/*D*_S_)/(*P*_N_/*P*_S_). Both *P*_N_ and *P*_S_ reflect a combination of neutral and deleterious alleles and thus represent an expected value for a neutral *D*_N/_*D*_S_ if no ancestral PS has occurred. If *FI *>* *1, then ancestral adaptation through beneficial nonsynonymous changes in the most recent common ancestor of *A. thaliana* and *A. lyrata* can be concluded to have occurred; alternately, if *FI *<* *1, then it implies that purifying selection on the ancestral lineage was the predominant selective force. For the 110 imprinted genes, we found that *D*_N_/*D*_S_ (1.139) approximated *P*_N_/*P*_S_ (1.196) with *FI = *0.952 (table 3) and conclude that there is no evidence of relaxed selective constraints. (We note that neither *D*_N_/*D*_S_ and *P*_N_/*P*_S_ ratios of these imprinted gene sets were biased by outliers; Daub et al. 2014). To further examine the recent selective pressures acting on *A. thaliana* imprinted genes, we also performed Direction of Selection (DoS) analysis which can produce more accurate estimates of selection, especially for highly conserved genes. In agreement with the results of the McDonald–Kreitman test, DoS analysis did not indicate any evidence of relaxed selective constraints (supplementary table S10*B*, Supplementary Material online) according to the Tarone and Greenland Neutrality Index (NI_TG_=1.237; table 3). Here, NI >1 indicates that negative selection is preventing fixation of harmful mutations.

**Table 3.** Calculations Derived from McDonald–Kreitman Analyses of Genes Regulated by Genomic Imprinting in the *Arabidopsis thaliana* Endosperm.

Parameter	Polymorphism		Divergence
Nonsynonymous substitutions (*D*_N_)	1,988		4,740
Synonymous substitutions (*D*_S_)	1,662		4,161
Ratio of nonsynonymous/synonymous (*D*_N_/*D*_S_) substitutions	1.196		1.139
Fixation Index (*FI*)^a^		0.952	
Expected Fixation Index (*eFI*)^b^		1.205	
Neutrality Index (NI_TG_)^c^		1.237	
α^d^		−0.210	

Note.—Values were derived from comparisons between 80 sequenced *A. thaliana* accessions, using *A. lyrata* as outgroup. Full gene-by-gene results from which these figures were derived are presented in supplementary table S7, Supplementary Material online.

a Observed fixation index, calculated according to *FI* = (*D*_N_/*D*_S_)/(*P*_N_/*P*_S_).

b Expected fixation index (*eFI*).

c The Tarone and Greenland Neutrality Index (NI_TG_).

d Proportion of fixed nonsynonymous mutations driven by fixed positive selection fixed in *A. thaliana*, α = (*FI−eFI*)/*eFI*.

**Fig. 5.**
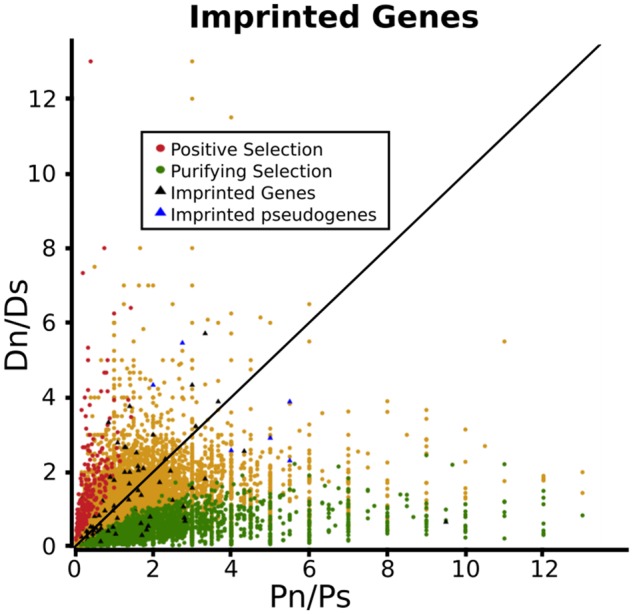
Distribution of *D*_N_/*D*_S_ and *P*_N_/*P*_S_ ratios for imprinted genes compared with all protein-coding genes in *Arabidopsis thaliana*. *X*-axis depicts *P*_N_/*P*_S_ ratios, *Y*-axis represents *D*_N_/*D*_S_ ratios. Green dots denote genes under purifying selection, red dots denote genes under positive selection, yellow dots denote genes under neutral evolution, black triangles denote *A. thaliana* imprinted genes, blue triangles denote pseudogenes with high *D*_N_/*D*_S_ and high *P*_N_/*P*_S_. No clustering was observed.

We also compared these values to those of the *A. thaliana* genome as a whole and found no evidence for imprinted genes differing from the genome-wide pattern (fig. 5). This suggests that the imprinted genes have been subject to similar selective processes as other genes since the divergence of *thaliana–lyrata* (supplementary fig. S3, Supplementary Material online): the same relative proportions showed patterns of PS (*D*_N_/*D*_S_ ≫ *P*_N_/*P*_S_), ancestral purifying selection (low *D*_N_/*D*_S_), neutrality (*D*_N_/*D*_S_∼*P*_N_/*P*_S_), or potential pseudogenization evidenced by relaxed selective constraint (high *P*_N_/*P*_S_ and high *D*_N_/*D*_S_) (Yang et al. 2011; Wang et al. 2012). In contrast to the PAML and HyPhy analysis of selection from before the *thaliana–lyrata* divergence, no difference was apparent between iMEGs and iPEGS (supplementary fig. S3, Supplementary Material online). Both McDonald–Kreitman and DoS analysis identified signatures of purifying selection on the same group of 13 genes (12% of the total, supplementary table S10*A* and *B*, Supplementary Material online) while six putative pseudogenes were discovered (5% of the total, supplementary table S11, Supplementary Material online): as expected, none of these showed any evidence of PS. As imprinted pseudogenes could potentially bias the overall analysis, their effect was assessed by comparing the baseline *FI* (0.952) to the expected fixation index (*eFI*, 1.205) determined from the expected contingency table values of *D*_N_, *D*_S_, *P*_N_, *P*_S_ for each of the 110 imprinted genes (Axelsson and Ellegren 2009). This higher *eFI* suggested population-level mutations were negatively correlated with purifying selection, presumably due to deleterious alleles segregating within the 80 accessions and supporting previous reports of high *P*_N_ values in *A. thaliana* (Huber et al. 2014). This is also important as relaxed selective constraints (evident from a high level of within-*A. thaliana* nonsynonymous changes) would have confounded our interspecies tests for positive selection, and because previous work has shown that the average effect of nonsynonymous changes in *A. thaliana* is slightly deleterious (Bustamante et al. 2002).

Comparison of the results of PAML and HyPhy analysis, McDonald–Kreitman tests and DoS demonstrates that the imprinted genes subject to positive selection in interspecies analysis using at least six genomes do not show any strong evidence of positive selection since the divergence of *A. thaliana* and *A. lyrata*. We conclude that genes with different evolutionary trajectories are regulated by genomic imprinting in *A. thaliana*, including some subject to pseudogenization while nonpseudogenized genes show signatures of ancestral PS with stronger signatures of PS predating the *thaliana*–*lyrata* split. Estimating the timing of these events with greater accuracy, and determining their effects in extant populations, will provide a basis for future determination of the selective pressures involved in the evolution of imprinted genes in plants.

## Discussion

Evolutionary trajectories of genes in mammals and angiosperms can be influenced by their association with tissues involved in maternal provisioning, creating the possibility for conflict over resource allocation and positive selection (PS) on the loci involved, among other molecular signatures (fig. 1). In this study, we have concentrated on the molecular signatures of conflict acting on coding sequences of imprinted genes in which alleles are expressed at different levels depending on whether they are maternally- or paternally derived (denoted iMEGs and iPEGs respectively; Köhler et al. 2012). The phenotypes associated with certain imprinted genes under PS in animals (*Igfr*) and plants (*AlMEDEA*) supports the possibility of conflict-driven PS (Spillane et al. 2007; Miyake et al. 2009; Wawrzik et al. 2010; McCole et al. 2011). However, we have previously demonstrated that there is no strict concordance between evidence of positive selection and imprinting status in mammals (O’Connell et al. 2010), and how conflict affects imprinted plant genes in general remains unknown.

In this study, we have performed a comprehensive ortholog-based analysis of selective pressures on genes subject to genomic imprinting in the seed endosperm of *A. thaliana* and have demonstrated signatures of elevated PS (tables 1 and 2; figs. 2 and 3; supplementary fig. S1, Supplementary Material online). To ensure these conclusions are robust, we have considered and accounted for the effects of possible endosperm-specific effects and of differences in gene age (fig. 4) and have accounted for potential confounding by genes expressed uniparentally from maternal tissues (supplementary fig. S2, Supplementary Material online). As approaches for inferring selection pressures may be limited by their own inherent assumptions, we took a multiple-methodology approach. For example, PAML makes the assumption that selective pressures do not change on the branches where it is inferred, while HyPhy allows branch-specific selection to change across all branches. We used two methodologies for our ortholog-based analyses (PAML and HyPhy) and for our analysis of extant *A. thaliana* populations (McDonald–Kreitman and Direction of Selection tests). In fact, the 30 imprinted genes founds to be under PS by PAML analysis were confirmed in every case confirmed as such by at least two HyPhy methods (supplementary tables S1 and S2, Supplementary Material online), while similar conclusions were derived from both McDonald–Kreitman and DoS approaches (table 3). We also note that it is not currently feasible to assess such changes at gene regulatory sequences across lineages, so our estimates for selection levels across loci, based as they are on coding-sequences alone, may in fact be underestimates.

It should be noted that some assumptions still remain within our analyses. For example, all *D*_N_/*D*_S_ based methods for estimating selective pressure variation from sequence data assume that *D*_S_ is a proxy for neutral evolution, that is, silent sites are not under selective pressure, even though we know, for example, that exon splice sites can be subject to selection to function the spliceosomal machinery (albeit mostly in intron-rich genomes; Warnecke et al. 2009). To control for this, we made use of nonimprinted controls, both from genome-wide data and from genes specifically expressed in the endosperm in which genomic imprinting occurs in flowering plants (supplementary table S4, Supplementary Material online). The robustness of the results from these analyses is furthermore supported by the robustness of the phylogeny used, which is uncontroversial (fig. 4; https://phytozome.jgi.doe.gov/pz/portal.html; last accessed July 2015), and on the number of species used in each alignment, which was set at a minimum of six, following experimentally determined best practice (Anisimova et al. 2001).

Combining together these analyses, and their comparison with relevant controls, we conclude that accelerated evolution and preferential tendency to PS are general features of imprinted genes in *A. thaliana*.

### Fixation of Selected Sites and Significance of Mating System

Extant plant lineages have undergone multiple transitions between self-fertilizing and out-crossing reproduction. It is expected that parental conflict will be minimized by increased levels of self-fertilization, which reduces or eliminates the genetic divergence between maternally- and paternally derived genomes (Haig 1997, 2013; Gehring and Satyaki 2017), as well as slightly reducing the efficacy of purifying selection across the genome (Payne and Alvarez-Ponce 2018). Consistent with this, previous investigations of the imprinted maternally expressed gene (iMEG) *MEDEA* found that *MEDEA* was under positive selection in the outcrossing Brassicaceae species, *Arabidopsis lyrata*, while its nonimprinted paralog *SWINGER* was not; but that neither gene was under positive selection in the largely inbreeding congener, *A. thaliana* (Spillane et al. 2007; Miyake et al. 2009). This was interpreted as a consequence of reduced genomic conflict due to inbreeding (Garnier et al. 2008; McKeown et al. 2013). The findings of our present study indicate that almost all of the positively selected sites are now fixed across populations in extant *A. thaliana* which may indicate that conflict has been reduced in this largely self-pollinated species: while the levels of outcrossing in *A. thaliana* can reach 18% in natural populations in exceptional cases, it is generally much lower (Bomblies et al. 2010).

The fixation of sites under positive selection in imprinted genes of *A. thaliana* is consistent with hypotheses that imprinting may in some cases be a relic of its outbreeding past (Brandvain and Haig 2005), perhaps because loss of imprinting to protect against deleterious recessive mutations only occurs very slowly (Wilkins and Haig 2003b). In other words, the signatures of selection detected by nonsynonymous changes to coding sequences retain evidence of past conflict even after any such equilibrium has been reached: our PAML analysis is in fact identifying sites which have changed under positive selection but are now at a stable equilibrium, and which no longer show signatures of such pressures in current populations (whether measured by McDonald–Kreitman tests or by Direction of Selection tests; supplementary tables S10 and S11, Supplementary Material online). Whether amino acid changes at these sites have also become fixed across other plant lineages with different levels of inbreeding would be an interesting test of this hypothesis, and will be possible to test empirically when once genomic data from multiple accessions of sufficient numbers of outcrossing and inbreeding plant species becomes available. It should also be noted that clonal interference arising from inbreeding is expected to marginally reduce the efficiency of selection across the genome (Neher et al. 2013) and potentially mask signatures of positive selection, although rates of neutral evolution at silent sites should not be affected (Good et al. 2014), provided that the beneficial alleles co-occur in the same period of selection. Therefore, clonal interference would mean tests for positive selection would be more prone to false negatives rather than false positives.

In addition, we have compared our rates of positive selection in imprinted loci to the genome-wide pattern for *A. thaliana*, which also adjusts for any potential confounding effects of inbreeding. Whether fixed or not, imprinted genes which have been under PS are likely to have been important for plant fitness and represent strong candidates for future functional investigations.

### Imbalance between Selective Pressures Acting on iMEGs and iPEGs

Imprinted genes in mammals can undergo different evolutionary trajectories (O’Connell et al. 2010; McCole et al. 2011). Our results from this study in plants demonstrate that differential selective pressures act on imprinted genes that are expressed from either the maternal or the paternal genomes. Specifically, iPEGs display higher *D*_N_/*D*_S_ values, and are significantly more likely to be subject to PS. This finding of asymmetric selection pressures on iPEGs versus iMEGs does not fit neatly with expectations of kin conflict which predict that any PS driven by intragenomic conflict should likely act on both genomes due to the mutual antagonism between the parents over resource allocation to the offspring, possibly on pairs of reciprocally imprinted genes encoding physically interacting offspring growth regulators (Moore and Haig 1991; Mills and Moore 2004).

Our identification of PS in iPEGs also lacks concordance with theories that propose that imprinting results from maternal-offspring coadaptation or cytonuclear coevolution as illustrated in figure 1*B* (Wolf and Hager 2006), in line with the lack of experimental support for this model (Haig 2013, 2014). Although coevolutionary scenarios can lead to rapid evolution of genes (Wolf and Brandvain 2014), both of these scenarios would be expected to preferentially affect iMEGs (assuming maternal cytonuclear inheritance). Nor is PS in iPEGs due to genome dosage effects in the endosperm, as the levels of positive selection for iPEGs are significantly higher than biallelically expressed endosperm genes (fig. 3). We can also rule out the possibility that PS in iPEGs could be an artifact of these genes being younger than iMEGs, because (1) there is no significant age difference between iPEGs and iMEGs, and (2) PS does not affect the more recently evolved iPEGs (figs. 2 and 5). We do note that levels of PS in the endosperm-expressed control set are slightly greater than the background control set (fig. 3*B*), which could indicate the existence of unreported iPEGs within this data set, or other causes related to the role of the endosperm in seeds. Finally, our results do not support an evolutionary scenario where imprinted genes arise as a result of pseudogenization following gene duplication (Wolff et al. 2011), as we could only identify six possible examples of this (fig. 2).

The finding that *A. thaliana* iPEGs are preferentially affected by PS compared with iMEGs provides an interesting parallel with the evolutionary flexibility of iPEGs observed in comparisons to *A. thaliana’s* sister species, *Arabidopsis lyrata*. Analysis of *A. lyrata* endosperm found that iPEGs were more highly expressed in *A. lyrata* than *A. thaliana*, while expression levels of iMEGs were more highly conserved (Klosinska et al. 2016). These changes were also associated with greater variation in CHG methylation and histone modification marks between at least some conserved iPEGs in the two species (Klosinska et al. 2016). Furthermore, a study in *Capsella rubella* showed that iPEGs display higher levels of nonsynonymous substitution, a possible indicator of PS (Hatorangan et al. 2016), suggesting that this pattern may not be restricted to the *Arabidopsis* genus either but may be a common feature of imprinting in, at least, the Brassicaceae. One possible explanation for the differences between selective pressures acting on iMEGs and iPEGs is that kin conflict more commonly involves interactions between iPEGs and genes expressed in maternal tissues such as the sporophytic seed coat (which are also involved in maternal provisioning; Orozco-Arroyo et al. 2015), rather than with iMEGs in the endosperm. This would lead to conflict that was indirect in nature, rather than involving physical interactions between antagonistic pairs of iMEGs and iPEGs (McVean and Hurst 1997). Intriguingly, an analysis of parental conflict in *A. lyrata* populations with different levels of outbreeding suggested that conflict involving indirect interactions between paternal factors and the female sporophyte (“the kinship model”) was favored in more self-fertile populations, while direct interactions between proteins encoded by imprinted genes in the endosperm tended to be lost as outcrossing reduced (Willi 2013). This would also fit with the discovery that genes which are strongly expressed in the seed coat of *A. thaliana* can also evolve under positive selection (Schon and Nodine 2017). We also note that antagonism between the developing endosperm and another maternal tissue, the nucellus, has been proposed as a key characteristic of seed development in *A. thaliana* (Xu et al. 2016). Analysis of the genetic interactions between maternal seed coat or nucellus with iPEGs which regulate seed size (such as *ADMENTOS*; Kradolfer et al. 2013) will therefore be required to clarify whether parental conflict occurs in *A. thaliana* and related species, and if so by what mechanism.

Further possible explanations for the differences in selective pressures acting on iMEGs and iPEGs could include differential breadth of expression patterns (including in somatic tissues) or wider interaction networks which could theoretically place iMEGs under greater constraints due to risk of pleiotropic interactions. Alternatively PS could also be due to so-called “arms races” between siblings that do not share the same paternal parent (Sadras and Denison 2009), which is more likely among paternally derived “patrigenes” than maternally derived “matrigenes” (Haig 2013). It has been shown that PS in flowering plants can be driven by prefertilization sexual conflict between male genomes during pollen tube competition (Gossmann et al. 2014), in a manner analogous to competition between animal sperm (Torgerson et al. 2002), such that positive selection at iPEGs could be triggered by conflict between the paternal genomes of endosperm tissues within seeds developing on the same plant (or in the same fruit). Paternal genetic variation is known to influence resource allocation in embryos by up to 10% in *A. thaliana* (House et al. 2010), which could be sufficient to drive conflict between paternal alleles. Finally, if this pattern was also conserved in monocots, it could explain reports that paternally derived expression-QTLs (eQTLs) have major roles in determining transcription levels in hybridized maize seed (Swanson-Wagner et al. 2009). Finally, the most active evolutionary signatures acting at iPEGs in different species of Brassicaceae (this study; Hatorangan et al. 2016; Klosinska et al. 2016), in which multiple shifts of mating system have occurred, could suggest that shifting patterns of paternal relatedness, and hence, patrigenic phenotypic optima for seed size, could lead to continual evolutionary pressure manifested in different ways, such as changes to transcription level, epigenetic marks, and changes to the nucleotide and amino sequence. More generally, models of imprinting and conflict suggest that matrigenes typically favor phenotypes intermediate to those favored by patrigenes and maternal alleles (Burt and Trivers 1998; Wilkins and Haig 2002, 2003a; Haig 2013), in which case, positive selection for conflict with maternal tissues would be stronger on paternally expressed imprinted genes than on maternally expressed ones. If so, the same trend might be expected to be common across seed plants: analysis of selective pressures acting on imprinted genes in a more distantly related group such as the cereals could be instructive in testing this hypothesis.

Given these different, and nonmutually exclusive possibilities, careful analysis of the functions of the genes and codons subject to PS will be needed to clarify the underlying impacts of the patterns we observe on the biology of the plant. Although experimental characterization for many genes has yet to be fully performed, we note that one of the iPEGs, we have identified to be under PS is *NRPD1a*, which encodes a subunit of RNA Pol IV, while other sRNA genes are not subject to PS (supplementary table S8, Supplementary Material online). RNA Pol IV is involved in control of transposable elements via RNA directed DNA methylation (RdDM) and has recently also been identified as a regulator of allelic dosage in the endosperm (Erdmann et al. 2017). Interestingly, the largest subunits of PolV (NRPE1), which is also implicated in the activity of 24-nt sRNAs in RNA-directed DNA methylation (RdDM), has also been reported to evolve rapidly through restructuring of intrinsically disordered repeats within its Argonaute-binding platform (Trujillo et al. 2016). In the case of NRPD1a, this subunit is involved in physically binding transposable elements including those expressed in maternal tissues in seeds (Mosher et al. 2009). Hence, it is possible that PS could be driven by conflict between paternally expressed proteins and maternally controlled transposable elements, or to interactions with the maternally derived genomes of the endosperm in the case of dosage control (Erdmann et al. 2017). Interestingly, *NRPD1a* does not appear to be an iPEG in *A. lyrata*, although two other genes encoding subunits of complexes involved in the RdDM pathway are (Klosinska et al. 2016). Further functional characterization of the positively selected subunits will be needed to distinguish these possibilities.

We note that positive selection has been reported from the iMEG *MEDEA* in the predominantly outcrossing *A. lyrata*, but that this selective pressure has been lost in the inbreeding *A. thaliana* lineage (Spillane et al. 2007). This lends further support to the hypothesis that positive selection persists between iPEGs and the maternal sporophyte but not between iPEGs and iMEGs during the transition to self-fertilization (Willi 2013). Analysis of signatures of selective pressure on the components of the FIS complex across multiple plant species will be essential for clarifying the effects of parental conflict in imprinting, endosperm development and speciation.

### Conclusions

The study of imprinted genes in both plants and mammals has identified examples of positive Darwinian selection (Spillane et al. 2007; O’Connell et al. 2010; Wawrzik et al. 2010). Our study demonstrates that while imprinted genes expressed in the endosperm of *Arabidopsis thaliana* are rapidly evolving due to positive selection, such positive selection is preferentially associated with imprinted paternally expressed genes (iPEGs). This raises the possibility that ongoing intragenomic conflicts between paternally expressed imprinted genes (iPEGs), or between iPEGs and genes functioning in the maternal sporophyte, could be evolutionary drivers and maintainers of imprinting in plants. The iPEG and iMEG genes we have identified under positive selection are involved in processes such as auxin biosynthesis (e.g., *YUCCA10*, *TAR1*) and epigenetic regulation involving small RNAs and chromatin remodelling (*NRPD1a*). Overall, our results identify the subset of imprinted genes, both iPEGs and iMEGs, which are strong candidates for having functional effects that are antagonistic with other molecular factors, in a manner that results in their evolution under positive selection.

## Materials and Methods

### Identification of Imprinted Genes and Orthologs

An *A. thaliana* imprinted gene set was compiled from a number of high-throughput expression screens (Gehring et al. 2011; Hsieh et al. 2011; McKeown et al. 2011; Wolff et al. 2011), supplemented by other studies (Vielle-Calzada et al. 1999; Kinoshita et al. 2004; Köhler et al. 2005; Jullien et al. 2006; Tiwari et al. 2008; Gehring et al. 2009; Gerald et al. 2009) to yield 140 high-confidence imprinted genes (supplementary table S1, Supplementary Material online). Orthologs were identified across 34 plant species for which assembled whole genome sequences were publically available (fig. 4). Peptide and CDS sequences for 32 species were downloaded from Phytozome v8.0 (Goodstein et al. 2012); *Cajanus cajan* sequences were accessed from (Varshney et al. 2012) and *Lotus japonicus* from the PlantGDB database (Dong et al. 2004). In all cases, the longest transcript was used as the representative transcript for each gene. To minimize the number of false positives and ensure tight clustering of genes families, we detected orthologous relationships between sequences using OrthoMCL (Li et al. 2003; Chen et al. 2007). We also chose to use maximum likelihood methods based on codon models of sequence evolution as these are considered to be more robust than alternative methods such as sliding window approaches (Schmid and Yang 2008). As the power of maximum likelihood methods increases with greater taxonomic representation and breadth (Anisimova et al. 2001), we considered only the 62 imprinted genes for which orthologous genes could be identified from at least six other species (in addition to *A. thaliana* itself). As controls, random sets of 100 genes were generated representing the entire *A. thaliana* genome, and a subset of endosperm-specific genes derived from (Belmonte et al. 2013) (supplementary table S4, Supplementary Material online). To ensure a valid comparison with the imprinted data set, only genes belonging to orthology clusters present in at least six other species (Anisimova et al. 2001) were included in these control sets.

### Multiple Sequence Alignments

Multiple sequence alignments for each gene family were constructed using MUSCLE (Edgar 2004) and MAFFT (Katoh and Toh 2008) and were compared in AQUA (Muller et al. 2010). RASCAL (Thompson et al. 2003) was used to refine the alignments and norMD (Thompson et al. 2001) was used to assess their quality. Alignments with a norMD score <0.6 were considered as low quality. Poorly aligned sequences were removed from alignments with norMD <0.6 and norMD was recalculated: if the norMD score subsequently increased to >0.6, the alignment was retained for further analysis. Nucleotide sequence alignments were generated for each family using the amino acid alignment and original nucleotide sequence files, using in-house software. Recombinant sequences were also removed identified using RDP3 (Martin et al. 2010) with two substitution-based methods—GENECONV (Sawyer 1989) and MaxChi (Smith 1992)—and two phylogenetic-based methods—BOOTSCAN (Martin et al. 2005) and SiScan (Gibbs et al. 2000). Sequences were considered as recombinant if a recombination event was significantly predicted by at least one substitution-based method *and* at least one phylogenetic-based method. The percentage of gaps in the alignments were calculated using TrimAL (Capella-Gutierrez et al. 2009) (-sgc option) and predicted sites of positive selection which overlapped with regions of poor alignment (gaps > 40%) were discarded.

### Tree Building

Models for protein sequence evolution were generated using modelgenerator (Keane et al. 2006). Phylogenetic trees were inferred using RAxML (Randomized Axelerated Maximum Likelihood) version 7.2.6 (Stamatakis 2006) with 1,000 bootstrap replicates and the rapid bootstrapping algorithm. The codeML analysis was run on all clades of interest for genes with >80 sequences in their orthology clusters (supplementary table S12*A*, Supplementary Material online) and on control genes from genome-wide and endosperm-expressed data sets (supplementary table S12*B*, Supplementary Material online).

### Selective Pressure Analysis

Selective pressure analysis was conducted using PAML version 4.4e (Yang 2007). Both lineage-specific models (Yang 1998; Yang and Nielsen 2002) and site-specific models (Yang and Nielsen 2002) were evaluated using likelihood ratio test (LRT). Sequences were considered to exhibit lineage-specific selective pressure if the likelihood ratio test for ModelA was significant in comparison to both ModelA null and M1Neutral, where M1Neutral is a neutral model that allows two site classes: ω_0_=0 and ω_1_=1. Model A assumes the two site classes are the same in both foreground and background lineages (ω_0_=0 and ω_1_=1) and ω_1_ was calculated from the data. Model A null is the null hypothesis for this model and allows sites to be evolving under either purifying selection, or to be neutrally evolving in the background lineages. For site-specific analyses, LRTs were conducted to compare models M7 and M8a with model M8. The test compared the neutral model M7, which assumes a β distribution for ω over sites and the alternative model M8 (β and ω), which adds an extra site class of positive selection. M8a is the null hypothesis of M8 where the additional category is neutral, that is, ω = 1. An automated CodeML wrapper (VESPA, Webb et al. 2017) was used to prepare all the codeML files, to parse the PAML output and perform the likelihood ratio test. After ML estimates of model parameters were obtained, we used two Bayesian approaches to infer the posterior probability of the positively selected sites: Bayes Empirical Bayes (BEB) and Naïve Empirical Bayes (NEB). BEB reduces the rate of false positives when analyzing small data sets and retains the power of NEB when analyzing large data sets (Yang and Nielsen 2002). Therefore, if NEB and BEB were both predicted the results from BEB were preferred.

### Use of HyPhy to Estimate Rates of Darwinian Selection

A second positive selection pressure analysis of genes which were predicted to be under positive selective pressure by PAML was conducted using HyPhy version 2.2.4 (Pond and Muse 2005). We employed the following three approaches from the HyPhy package: FEL (Fixed effects Likelihood), SLAC (Single-Nucleotide Ancestor Counting), and MEME (Mixed Effects Model of Evolution). FEL tests for both positive and negative selection per individual site, and can identify individual sites that have undergone pervasive diversifying selection while SLAC is an approximate method similar to FEL (Kosakovsky Pond and Frost 2005). We also applied the MEME model from the HyPhy package which tests for episodic selection at individual sites and on specific branches: MEME does not assume that the strength and direction of selection is constant across all lineages (Murrell et al. 2012). Only sites resolved as being under PS by at least two methods were considered confirmed by HyPhy.

### Tests Including Population-Level Variation

*Arabidopsis lyrata* orthologs of 140 imprinted *A. thaliana* genes were identified using reciprocal best hits (RBH) of which 110 were also derived as the best hits of the *A. thaliana* genes in reciprocal BLAST. *Arabidopsis thaliana* and *A. lyrata* CDS were aligned as described earlier. About 80 accession SNP data for *A. thaliana* was downloaded from the 1001 genome project (http://1001genomes.org/data/MPI/MPICao2010/releases/current/genome_matrix; last accessed April 2015) and SNPs mapped to the reference genome using a custom-made python script. McDonald–Kreitman tests were performed on each imprinted gene using a python script that uses egglib library to calculate *D*_N_, *D*_S_, *P*_N_, and *P*_S_ values and calculated the ratio using Fisher’s exact test. Fixation indices (*FI*) were determined as *FI*= (*D*_N_/*D*_S_)/(*P*_N_/*P*_S_) with expected fixation index (*eFI*) calculated as reported previously (Axelsson and Ellegren 2009). Genes with zero *D*_N_/*D*_S_ and *P*_N_/*P*_S_ were not considered for *FI* calculations. Direction of selection (*DoS*) (Stoletzki and Eyre-Walker 2011) was calculated using *D*_N_/(*D*_N_+*D*_S_)−*P*_N_/(*P*_N_+*P*_S_); the Tarone and Greenland Neutrality Index (NI_TG_) was calculated using the Distribution of Fitness Effect (DoFE) package.

## Supplementary Material

Supplementary data are available at *Molecular Biology and Evolution* online.

## Supplementary Material

Supplementary_Material_msz063
