# Supplementary material for: Paternally Expressed Imprinted Genes under Positive Darwinian Selection in Arabidopsis thaliana
Source: Mol Biol Evol. 2019 Mar 26;36(6):1239–53. doi: 10.1093/molbev/msz063 (PMC6526901; doi:10.1093/molbev/msz063)
Supplement: Supplementary_Material_msz063 [file supplementary_material_msz063.zip › Supplementary Tables S5 S6 S8 S11_final.pdf]

Supplementary Tables S5, S6, S8 & S11

Supplementary Table S5

A: Sites in imprinted genes identified to be under lineage-specific positive selection.

Gene ID	Imprinting Status	Putative function of protein	Positively Selected Sites	P-value for positively selected sites
AT1G20910	iPEG	ARID/BRIGHT DNA-binding domain-containing protein	23, 84, 93, 97*, 113, 114, 123, 139*, 147, 153, 169, 179*, 183, 195*, 198*, 234, 255, 257, 269, 270, 308*, 317, 322, 335, 339, 346*, 347, 368, 372, 384, 386, 392	32 > 0.5, 7 > 0.95, 2 > 0.99
AT1G42470	iMEG (low-confidence)	ATNPC1 - 1, NIEMANN-PICK DISEASE TYPE C1-1	42, 338, 622, 1024, 1187, 1210, 1221, 1240*	8 > 0.5, 1 > 0.95, 0 > 0.99
AT1G47490	iPEG	RNA-binding protein 47C	20, 115, 125, 173, 231, 321, 392	7 > 0.5, 0 > 0.95, 0 > 0.99
AT1G48910	iPEG	YUCCA 10	153*, 362	2 > 0.5, 1 > 0.95, 1 > 0.99
AT1G49600	iPEG	RNA-binding protein 47A	105, 349*	2 > 0.5, 1 > 0.95, 1 > 0.99
AT1G54280	iMEG	AMINOPHOSPHOLIPID ATPASE 6	965*	1 > 0.5, 1 > 0.95, 0 > 0.99

AT1G55050	iMEG	Protein of unknown function	26, 40, 47, 54, 173*, 179, 183, 189, 246, 272, 275, 277, 294, 306, 311, 337, 341, 348, 363*, 368, 392, 396*, 401, 433, 449, 450, 451*, 453, 470, 484, 504, 566, 567, 579, 580*, 581, 582*, 583*, 584*, 608, 611, 658, 659, 661, 662, 665, 669, 672, 673, 675, 676, 677, 683, 684, 695, 696, 697, 698*, 699*, 700, 701, 702*, 703, 704, 705, 706*, 707, 709, 710, 711, 712, 716, 717*, 718, 719, 720, 721, 722, 723, 724*, 726*, 729*, 731, 732, 735, 739, 740, 741, 742, 744, 745, 748, 749, 750, 752, 755, 756, 758, 759, 760, 761, 762, 763, 764*, 765, 766, 768, 769, 770, 771, 780, 806, 813, 846, 854*, 855, 856, 857, 858, 859, 861, 862, 865, 866, 870	125 > 0.5, 18 > 0.95, 2 > 0.99
AT1G63020	iPEG	NRPD1a	77, 201, 932, 1177, 1287	5 > 0.5, 0 > 0.95, 0 > 0.99
AT1G67820	iPEG	Protein phosphatase 2C family protein	239, 404*, 410*	3 > 0.5, 2 > 0.95, 1 > 0.99
AT1G76820	iMEG (low-confidence)	eukaryotic translation initiation factor 2 (eIF-2) family protein	170, 172, 173, 176, 186, 227, 234, 238, 239, 240*, 241, 250, 342*, 422, 423, 439, 441*, 455, 459, 929, 1017, 1047, 1101, 1145	24 > 0.5, 3 > 0.95, 0 > 0.99
AT2G40520	iPEG	Nucleotidyltransferase family protein	5, 29, 53, 62, 63, 68, 77, 82, 100, 102, 103, 121, 124, 128, 132, 135, 145, 148, 179*, 184, 233, 234, 246, 285*, 287, 330, 333, 356, 357*, 381*, 386, 395, 423, 427, 431, 432, 442, 478, 479	39 > 0.5, 4 > 0.95, 2 > 0.99
AT5G28300	iPEG	GT-2LIKE PROTEIN, GT2L	72, 575, 576, 578, 579, 581	6 > 0.5, 0 > 0.95, 0 > 0.99
AT5G53150	iPEG	DNAJ heat shock N-terminal domain-containing protein	49, 62, 115, 150, 439, 442, 509, 526, 624	9 > 0.5, 0 > 0.95, 0 > 0.99

*** Positively selected sites with posterior probability > 0.95 are marked with asterisk (*)**

B: Sites in imprinted genes identified to be under site-specific positive selection.

Gene ID	Imprinting Status	Putative function of protein	Positively Selected Sites	P-value of positive selected sites
AT1G20910	iPEG	ARID/BRIGHT DNA-binding domain-containing protein	7, 8, 11*, 12, 17, 19, 22, 23*, 24, 25, 27, 28*, 35, 39, 43, 48, 49, 50, 52, 53, 54, 57, 62, 65, 69, 71, 72, 73, 75, 76*, 398	31 > 0.5, 4 > 0.95, 3 > 0.99
AT1G23320	iPEG	TRYPTOPHAN AMINOTRANSFERASE RELATED 1	5, 8*, 11*, 12, 14, 15*, 16, 17, 18, 20*, 382, 383*	12 > 0.5, 5 > 0.95, 0 > 0.99
AT1G42470	iMEG (low confidence)	ATNPC1 - 1, NIEMANN-PICK DISEASE TYPE C1-1	299, 315, 1245, 1254, 1260, 1261	6 > 0.5, 1 > 0.95, 0 > 0.99
AT1G48910	iPEG	YUCCA 10	379*	1 > 0.5, 1 > 0.95, 1 > 0.99
AT1G55050	iMEG	Protein of unknown function	83*, 84*, 93, 98, 103, 112, 396, 453, 468, 473, 474, 477, 481, 484, 488*, 517, 518, 519*, 520, 525*, 527, 549*, 625, 626, 630, 631, 658, 660, 661, 672, 673, 676, 703, 704, 748, 750, 752, 753, 754, 755*, 756*, 758, 759*, 764, 765, 767*, 769*, 771, 836, 838, 840, 843, 847, 858, 860, 865, 866, 867, 868, 870	60 > 0.5, 11 > 0.95, 1 > 0.99
AT1G62660	iMEG	Glycosyl hydrolases family 32	14, 62, 63, 64, 65*, 66, 86*	7 > 0.5, 2 > 0.95, 0 > 0.99
AT1G67820	iPEG	CYTOKININ INDUCED ROOT CURLING 1 / TRYPTOPHAN AMINOTRANSFERASE OF ARABIDOPSIS 1 / WEAK ETHYLENE	30	1 > 0.5, 1 > 0.95, 1 > 0.99

		INSENSITIVE 8		
AT1G70560	iPEG	Protein of unknown function	3, 10, 11, 13, 16, 18*, 19*, 385, 386*	9 > 0.5, 3 > 0.95, 0 > 0.99
AT1G76250	iMEG (low confidence)	Protein of unknown function	54, 74, 77, 78, 79, 80	6 > 0.5, 0 > 0.95, 0 > 0.99
AT2G40520	iPEG	Nucleotidyltransferase family protein	30, 50, 51, 291, 346, 356, 371, 403*, 408, 410, 411, 412, 415, 417, 418, 419, 424, 426, 427, 428, 430*, 431, 432, 433, 435, 452, 453, 470, 475, 478*, 479*, 481, 484, 485, 486, 488, 489, 491, 492, 493, 494, 495, 496, 497*, 498*, 500, 502	47 > 0.5, 6 > 0.95, 1 > 0.99
AT3G08040	iPEG	FERRIC REDUCTASE DEFECTIVE 3	10, 14, 109, 112, 114, 116, 134, 136, 137, 139, 140, 141, 143, 144, 145, 147, 148, 149, 150, 151, 152, 153, 154, 156, 158, 160, 213, 291, 520, 521	30 > 0.5, 5 > 0.95, 1 > 0.99
AT3G14205	iPEG	SAC2, SUPPRESSOR OF ACTIN 2	5, 9, 10, 12, 14, 17, 450	7 > 0.5, 0 > 0.95, 0 > 0.99
AT3G26590	iMEG	MATE efflux family protein	7*, 15, 16, 18*, 19, 20*, 372, 491, 492*, 494*, 495, 496*, 498, 499*	14 > 0.5, 7 > 0.95, 5 > 0.99
AT3G45090	iPEG	P-loop containing nucleoside triphosphate hydrolases superfamily protein	20, 76, 78, 82, 83, 87, 88, 91, 623*, 624*, 629*, 636, 638, 685	14 > 0.5, 3 > 0.95, 1 > 0.99
AT4G18650	iMEG	Protein of unknown function	228, 232	2 > 0.5, 0 > 0.95, 0 > 0.99
AT4G20800	iPEG	FAD-binding Berberine family protein	4, 5, 20*, 294, 295*, 317, 363, 434, 436*, 493, 526, 527	12 > 0.5, 3 > 0.95, 1 > 0.99
AT4G26140	iMEG	BETA-GALACTOSIDASE 12	2, 4, 5*, 6, 7, 9, 10, 497, 518*, 553, 599, 630*	12 > 0.5, 3 > 0.95, 1 > 0.99
AT5G03370	iMEG	Acylphosphatase family	33, 36, 37, 48, 60, 64, 77	7 > 0.5, 1 > 0.95, 0 > 0.99

AT5G11460	iPEG	Protein of unknown function	6, 182, 254, 265	4 > 0.5, 0 > 0.95, 0 > 0.99
AT5G28300	iPEG	GT-2LIKE PROTEIN, GT2L	228*, 233, 248, 382, 383, 384, 387, 406, 407, 408, 415, 424, 426*, 427, 428, 429*, 430, 440, 571, 576, 577*, 578*, 579*, 588, 592, 596	26 > 0.5, 6 > 0.95, 3 > 0.99
AT5G42670	iPEG	Agenet domain-containing protein	45, 61, 63, 96, 161, 167, 212, 214, 226, 242, 273	11 > 0.5, 0 > 0.95, 0 > 0.99
AT5G43780	iPEG	Sulfate adenylyltransferase	9, 11, 12, 13*, 14, 15, 17*, 19, 20*, 21*, 22*, 23, 24*, 25*, 26*, 27*, 28, 29*, 32, 33*, 34*, 36*, 37, 43*, 46*	25 > 0.5, 15 > 0.95, 5 > 0.99
AT5G53150	iPEG	DNAJ heat shock N-terminal domain-containing protein	431, 439, 695	3 > 0.5, 0 > 0.95, 0 > 0.99
AT5G54650	iMEG	Formin homologue 5 (AtFH5)	14, 57, 176, 177, 189, 191, 202, 313, 323, 336, 363, 432, 492	13 > 0.5, 0 > 0.95, 0 > 0.95

*** Positively selected sites with posterior probability > 0.95 are marked with asterisk (*)**

Supplementary table S6. Percentage of the lineage-specific positively selected sites (posterior probability >0.95) that have reached fixation in the 80 sequenced *A. thaliana* accessions, ranked by posterior probability. Amino acids belonging to genes with no *A. lyrata* ortholog present in the tree (either not present or discarded from alignments due to presence of recombination) are marked with 'NA'.

Posterior probability	*Ath* amino acid	% conservation of sites in *Ath* 80 accessions	*Aly* amino acid
1	S	100	E
0.997	I	100	R
0.996	N	100	S
0.996	V	100	P
0.996	K	100	S
0.995	C	100	H
0.994	I	100	P
0.994	R	100	E
0.993	S	100	K
0.993	P	100	K
0.992	G	96.25	P
0.992	V	100	N
0.992	K	100	D
0.992	K	100	R
0.991	K	100	G
0.99	E	100	P
0.99	D	100	Q
0.989	Q	100	V
0.988	K	100	R

0.986	R	100	K
0.984	N	100	E
0.984	G	100	D
0.982	N	100	A
0.982	P	100	K
0.982	E	100	C
0.981	V	100	T
0.979	K	100	-
0.978	A	100	NA
0.978	F	92.5	Q
0.976	D	100	T
0.976	A	100	E
0.976	Y	100	NA
0.975	S	100	F
0.975	A	100	K
0.975	S	100	R
0.974	E	100	P
0.974	P	100	L
0.974	S	100	NA
0.973	Y	100	-
0.973	D	100	S
0.971	T	100	D
0.971	C	100	A
0.971	Y	85	P
0.969	A	100	-
0.969	N	100	Q
0.969	K	100	R

0.966	L	100	A
0.963	K	100	D
0.962	E	100	N
0.962	I	100	C
0.961	R	100	N
0.961	T	98.75	L
0.961	N	100	NA
0.96	K	100	R
0.957	G	100	E
0.957	L	100	-
0.956	S	100	N
0.955	K	100	R
0.954	L	100	NA
0.953	E	83.75	I
0.952	R	100	R
0.951	D	100	K
0.951	E	100	T
0.95	L	100	NA

Supplementary Table S8.

Gene name	Protein	Function	Reference	Positive selection
**At1g63020**	**NRPD1A**	**Core subunit of Pol IV**	**iPEG (Gerhing et al., 2011)**<br>**Kanno et al., 2005; Eamons et al., 2008.**	**Lineage specific PDS acting on five amino acids:**<br>**69 – within the RNA polymerase Rpb1, domain 1;**<br>**160 – in the same domain, very close the C-terminal end;**<br>**878 – one of 11 amino acids comprising the putative Rpb1 (NRPD1)-Rpb2 interaction site on conserved domain RNAP_IV_NRPD1_C, and within the DNA binding cleft of this domain;**<br>**1104 – within a later part of the DNA binding cleft of the same domain Rbp1 domain C;**<br>**1270 – at end of Rbp1 domain C.**
At2g40030	NRPD1B	Core subunit of Pol V	Haag & Pikaard, 2011	Absent
At3g23780	NRPD2A	Core subunit of Pol IV and Pol V	"	Absent
At3g18090	NRPD2B	"	"	Absent
At4g11130	RDR2	Interacts with NRPD1a (Pol IV)	"	Absent
At3g42670	CLASSY1	"	"	Absent
At2g30280	DMS4	"	"	Absent
At2g27040	AGO4	Downstream of NRPD1a in sRNA production	"	Absent

At3g43920	DCL3	"	"	Absent
At4g20910	HEN1	"	"	Absent
At3g22680	RDM1	Interacts with NRPD1b (Pol V)	Haag & Pikaard, 2011	Absent
At3g48670	IDN2	"	"	Absent
At2g16390	DRD1	"	"	Absent
At3g49250	DMS3	"	"	Absent
At5g04290	KTF1	"	"	Absent
At5g14620	DRM2	"	"	Absent
At1g01040	DCL1	Interacts with NRPD1a in pathogen response	Katiyar-Agarwal et al, 2006	Absent
At1g09700	HYL1	"	"	Absent
At5g23570	SGS3	"	"	Absent
At3g49500	RDR6	Maintains silencing at PolIV-regulated siRNAs	Haag & Pikaard, 2011	Absent
At5g66750	DDM1	Maintains sRNA-mediated silencing	"	Absent
At4g13940	HOG1	"	"	Absent
At3g54220	SCR	Interacts with NRPD1a in Self-Incompatibility response	Strickler et al., 2013	Absent
	SRK	"	"	Absent

Supplementary Table S11: High Dn/Ds and High Pn/Ps of *A. thaliana* imprinted genes identified as pseudogenes.

Gene ID	Dn/Ds	Pn/Ps	Mutation type	Number of accessions with broken gene models
AT4G29570	5.46	2.75	PREMATURE	1
AT3G57250	2.30	5.50	FRAMESHIFT	14
AT1G11810	2.91	5.00	FRAMESHIFT	31
AT1G61090	4.33	2.00	FRAMESHIFT	3
AT1G07690	2.58	4.00	FRAMESHIFT	1
AT1G20730	3.88	5.50	FRAMESHIFT	8
