# Supplementary figures and images for: Paternally Expressed Imprinted Genes under Positive Darwinian Selection in Arabidopsis thaliana
Source: Mol Biol Evol. 2019 Mar 26;36(6):1239–53. doi: 10.1093/molbev/msz063 (PMC6526901; doi:10.1093/molbev/msz063)

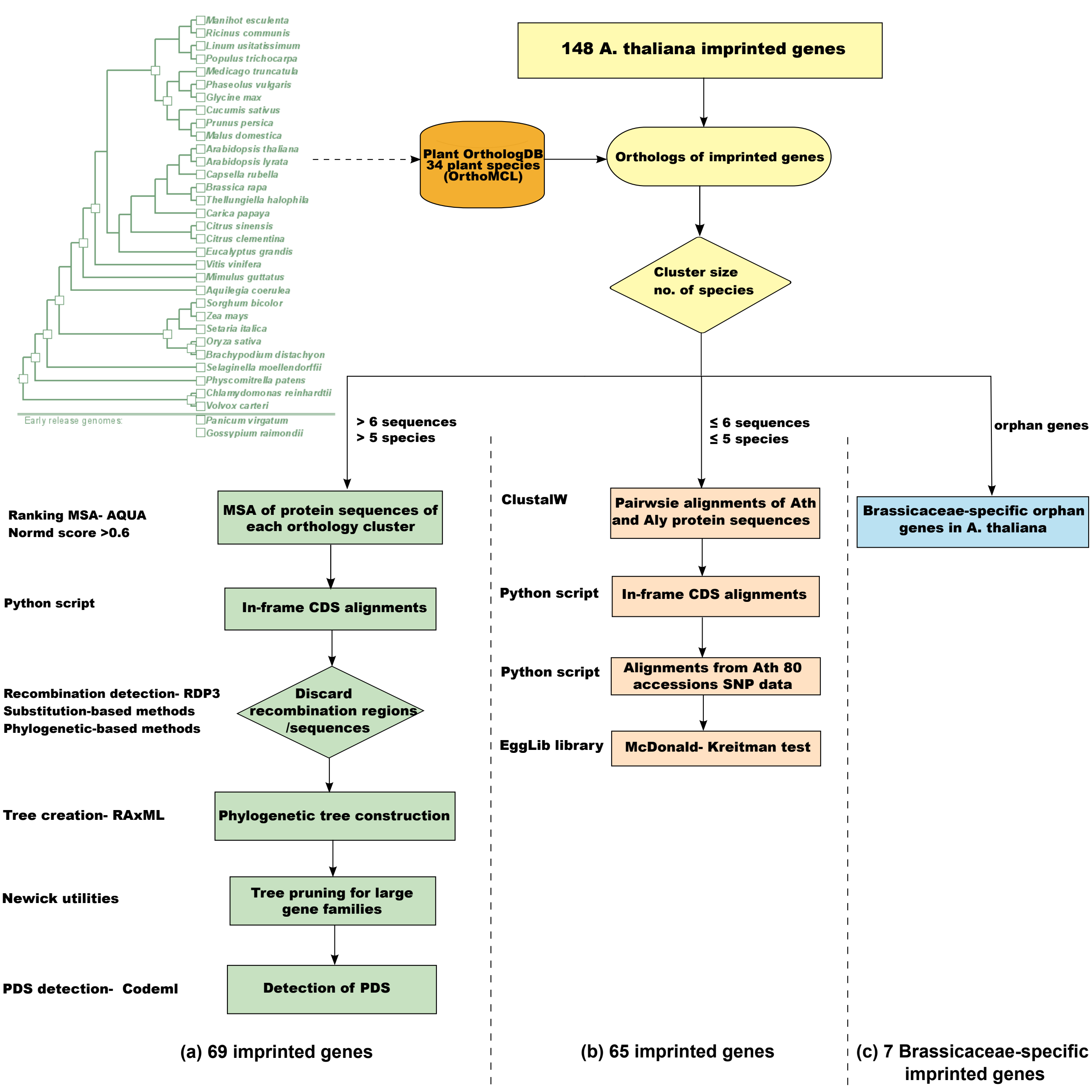

Supplement: Supplementary_Material_msz063 [file supplementary_material_msz063.zip › Supplementary Fig S1.pdf]

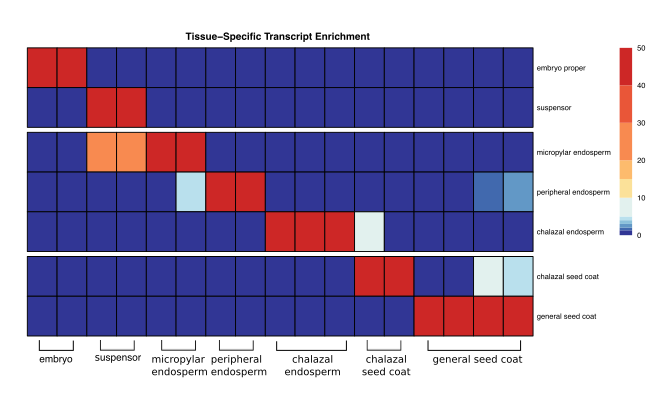

Supplement: Supplementary_Material_msz063 [file supplementary_material_msz063.zip › Supplementary Fig S2.tif]

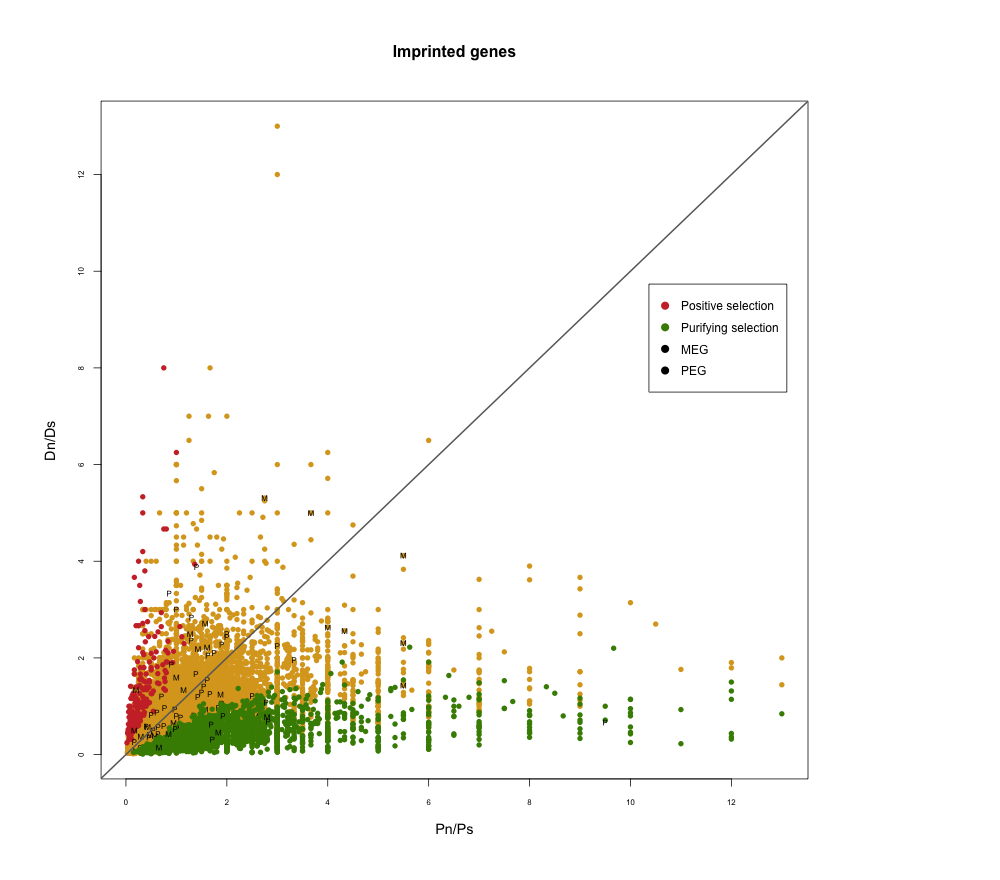

Supplement: Supplementary_Material_msz063 [file supplementary_material_msz063.zip › Supplementary Fig S3.tiff]
